# Drug Repurposing as a Broad-Spectrum Strategy Against Coronaviruses: Frontiers in Mechanisms and Clinical Translation

**DOI:** 10.3390/v18070804

**Published:** 2026-07-21

**Authors:** Shuai Du, Yue Wang, Chao Liu, Yiming Han, Rui Zong, Shen Wang, Wenjuan Du, Linyang Yu, Yongtao Li

**Affiliations:** 1 College of Veterinary Medicine, Henan Agricultural University, Zhengzhou 450046, China; dudu20249@163.com (S.D.); yuewang0124@163.com (Y.W.); 18739477037@163.com (C.L.); biohym@163.com (Y.H.); 17658403228@163.com (R.Z.); shengwang@henau.edu.cn (S.W.); wenjuandu111@163.com (W.D.); 2 International Joint Research Center of National Animal Immunology, College of Veterinary Medicine, Henan Agricultural University, Zhengzhou 450046, China; 3 Ministry of Education Key Laboratory for Animal Pathogens and Biosafety, Henan Agricultural University, Zhengzhou 450046, China; 4 Longhu Laboratory of Advanced Immunology, Zhengzhou 450046, China

**Keywords:** coronaviruses, broad-spectrum antiviral, drug repurposing, human–veterinary dual-track

## Abstract

Coronaviruses (family *Coronaviridae*) are enveloped, positive-sense, single-stranded RNA viruses with broad host adaptability. They transmit across species among humans, livestock, companion animals, and wildlife, eliciting a disease spectrum ranging from mild respiratory and gastrointestinal symptoms to fatal multi-organ failure, thereby posing significant challenges to global health. In this context, drug repurposing has emerged as a practical strategy for rapidly identifying broad-spectrum antivirals against emerging and re-emerging coronaviruses. Using coronaviruses as a paradigm, this review systematically summarizes research advances and mechanistic insights into the repurposing of existing drugs against coronaviruses. Simultaneously, we dissect core challenges including species-specific pharmacokinetic disparities, insufficient inter-genera conservation of viral targets, and systemic barriers between human and veterinary drug regulatory frameworks and propose innovative solutions encompassing AI-driven cross-species drug prediction, next-generation cross-species infection models, and a “human-veterinary dual-track” collaborative research and development system. Collectively, this review highlights the promise of drug repurposing as a broad-spectrum antiviral strategy and provides a translational perspective for the development of cross-species anti-coronavirus therapeutics.

## 1. Introduction

### 1.1. Overview of Cross-Species Coronaviruses

Since coronaviruses were first isolated from chickens in 1937, nearly 90 years of research have revealed these viruses as paradigm examples of cross-species emergence. They are named “coronavirus” because the spike proteins on the viral envelope are radially arranged under electron microscopy, giving the virus a crown-like appearance [1]. Taxonomically, coronaviruses are enveloped positive-sense single-stranded RNA viruses with genomes of 22–36 kilobases, representing the largest known group of RNA viruses [2]. Coronaviruses exhibit an exceptionally broad host range, including humans, other mammals, and birds, and commonly cause multisystem diseases, with predominant manifestations in the respiratory, digestive, and nervous systems [3,4]. According to the International Committee on Taxonomy of Viruses (ICTV), the family *Coronaviridae* is divided into four genera: *Alphacoronavirus, Betacoronavirus, Gammacoronavirus and Deltacoronavirus*, which differ significantly in host tropism, genetic evolution, and pathogenicity [2]. Currently, seven coronaviruses are known to infect humans: HCoV-229E and HCoV-NL63 belong to *Alphacoronavirus*, while HCoV-OC43, HCoV-HKU1, MERS-CoV, SARS-CoV, and SARS-CoV-2 belong to *Betacoronavirus*. Compared with the other four human coronaviruses, SARS-CoV, MERS-CoV, and SARS-CoV-2 cause respiratory diseases characterized by high transmissibility, severe pathogenicity, and wide prevalence, which may progress to pulmonary failure and death in severe cases [5,6,7]. Major animal coronaviruses include porcine epidemic diarrhea virus (PEDV), infectious bronchitis virus (IBV), canine coronavirus (CCoV), and feline coronavirus (FCoV), among others [8]. In addition, several coronaviruses identified across different species show potential for cross-species transmission [9].

**Figure 1:**
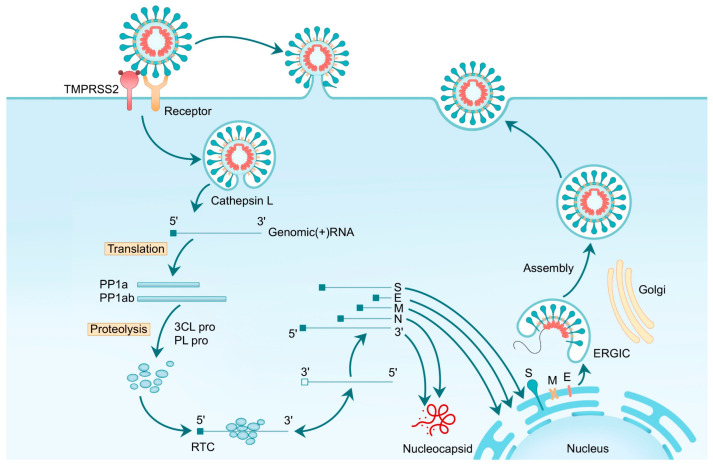
Schematic overview of the coronavirus replication cycle in host cells. The diagram illustrates the full course of coronavirus infection: spike (S) protein-mediated entry, polyprotein translation and cleavage by 3-chymotrypsin-like protease (3CLpro) and papain-like protease (PLpro), replication-transcription complex RTC formation, genome replication, structural protein trafficking, and mature virion assembly and release.

The genomes of these four genera contain multiple open reading frames (ORFs) encoding various structural and non-structural proteins. ORF1a and ORF1b occupy the majority of the genome, encoding viral replicase polyproteins; the remaining ORFs encode structural proteins, including spike (S), envelope (E), membrane (M), and nucleocapsid (N) proteins, as well as several accessory proteins. The spike (S) protein on the virion surface is critical for host recognition and entry: the S1 subunit binds host cell receptors (e.g., human angiotensin-converting enzyme 2 (ACE2) for SARS-CoV-2, feline aminopeptidase N for FIPV, and chicken sialic acid receptors for IBV), while the S2 subunit mediates fusion between the viral envelope and the host cell membrane [10,11,12]. The coronavirus S protein is highly correlated with viral cell tropism, tissue tropism, and cross-species transmission potential [13]. Following host cell entry, the viral genome is first translated into polyproteins pp1a and pp1ab encoded by ORF1a/1b, which are subsequently cleaved by virally encoded 3C-like protease (3CLpro/Mpro) and papain-like protease (PLpro) into 15 or 16 non-structural proteins (nsps). These processed nsps assemble on double-membrane vesicles (DMVs) to form the replication-transcription complex (RTC), which then initiates viral genome replication and generates subgenomic mRNAs through discontinuous transcription for translation of structural proteins S, M, E, N, and accessory proteins [14,15]. The M, S, and E proteins are trafficked through the endoplasmic reticulum to the Golgi apparatus, where they assemble with the N protein-encapsidated viral genome to form virions. The assembled virions are then released from host cells via exocytosis, generating infectious mature virions [16,17] (Figure 1).

Enzymes and proteins involved in the viral replication cycle are all potential targets for anti-coronavirus drugs. Current research on anti-coronavirus therapeutics mainly focuses on the spike (S) protein, RNA-dependent RNA polymerase (RdRp), 3CLpro, and PLpro. Together with host targets such as receptors, proteases, and immune pathways, these viral components constitute the core nodes for pharmacological intervention [18,19]. For example, 3CLpro exhibits highly conserved structural features and substrate specificity, and its Cys145–His41 catalytic dyad represents an ideal broad-spectrum antiviral target [20,21,22]. Meanwhile, the conserved domains of RdRp (e.g., finger, palm, and thumb subdomains) provide binding sites for nucleoside analogues [23,24]. PLpro harbors a conserved catalytic domain among coronaviruses, and its functions in polyprotein maturation and suppression of host innate immunity render it a valuable broad-spectrum antiviral target [25]. Host receptors (e.g., ACE2, heparan sulfate proteoglycans (HSPGs)) and host proteases (e.g., transmembrane protease serine 2 (TMPRSS2), furin protease, cathepsin L protease (Cathepsin L)) have also emerged as critical intervention targets, with related drug development progressing rapidly [26,27,28].

### 1.2. Epidemiological Characteristics and Public Health Risks of Cross-Species Transmission of Coronaviruses

Coronaviruses (CoVs) exhibit remarkable host adaptability in terms of genetic diversity, receptor utilization, and adaptive mutations, enabling frequent cross-species transmission. Their cross-species transmission primarily relies on the recognition and binding of viral spike proteins to host cell receptors (e.g., aminopeptidase N (APN), ACE2), and adaptive mutations can enhance viral infectivity in new hosts [13,29,30]. Epidemiological evidence has shown that cross-species transmission of coronaviruses has repeatedly posed severe threats to human health. SARS-CoV, which caused the SARS outbreak in 2003, is believed to originate from *Rhinolophus sinicus* (Chinese horseshoe bats) and was transmitted to humans via civets as intermediate hosts [31,32,33]. As for MERS-CoV, serological evidence, phylogenetic inference, and live virus isolation collectively implicated dromedary camels as its primary animal origin [34,35,36]. The zoonotic origin of SARS-CoV-2, which emerged in 2019, remains unclear [37,38]. In addition to threatening human health, coronaviruses are equally devastating among animal populations, inflicting immeasurable losses on global companion animal health and the livestock industry: PEDV and swine acute diarrhea syndrome coronavirus (SADS-CoV) can cause mass mortality in swine herds, with piglet mortality exceeding 80% [39,40]; feline infectious peritonitis virus (FIPV) infection in cats is almost invariably fatal in the absence of targeted antiviral therapy [41]; and infectious bronchitis virus (IBV) can reduce egg production in laying hens by 5% to 50% [42].

Notably, several animal coronaviruses have demonstrated the potential to infect humans. In 2021, porcine deltacoronavirus (PDCoV) was identified in plasma samples from three Haitian children with acute undifferentiated febrile illness [43]. In the same year, a novel canine coronavirus was isolated from hospitalized patients with pneumonia in Malaysia [44]. In 2025, bat-derived HKU5-CoV-2 was found to efficiently utilize human ACE2 receptors for cell entry and replicate robustly in human respiratory and intestinal organoids. Its receptor-binding domain (RBD) shares a similar ACE2 binding footprint with HCoV-NL63, indicating that bat coronaviruses may breach species barriers through diverse pathways [45]. Beyond coronaviruses, multiple zoonotic RNA viruses adopt convergent evolutionary strategies to broaden host ranges and trigger recurrent livestock epidemics, such as the Getah virus, whose nationwide epidemiological spread and adaptive evolution in China have been systematically characterized [46]. Such pervasive cross-species threats fully demonstrate the significance of drug repurposing for emergency responses to unexpected viral epidemics.

**Figure 2:**
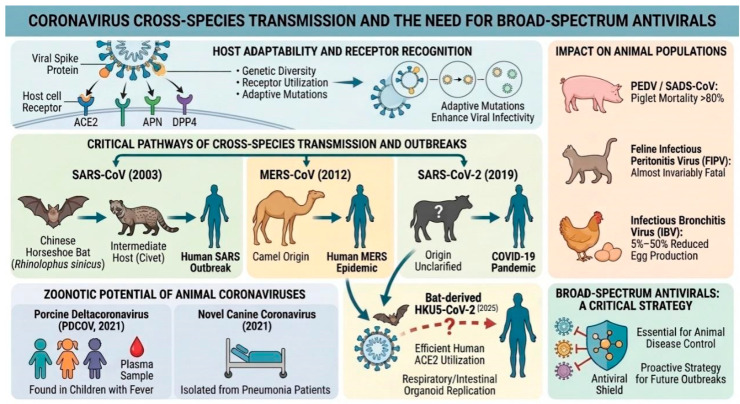
Coronavirus cross-species transmission and broad-spectrum antiviral strategies. *Coronaviruses* achieve host adaptability through genetic diversity, receptor utilization (angiotensin-converting enzyme 2 (ACE2), aminopeptidase N (APN), dipeptidyl peptidase 4 (DPP4)), and adaptive mutations. Documented zoonotic transmissions include severe acute respiratory syndrome coronavirus (SARS-CoV) (2003, bat-civet-human), Middle East respiratory syndrome coronavirus (MERS-CoV) (2012, camel-human), and severe acute respiratory syndrome coronavirus 2 (SARS-CoV-2) (2019). Animal coronaviruses cause substantial veterinary and economic impacts, including porcine epidemic diarrhea virus (PEDV)/swine acute diarrhea syndrome coronavirus (SADS-CoV) (>80% piglet mortality), feline infectious peritonitis virus (FIPV) (near-universal fatality), and infectious bronchitis virus (IBV) (5–50% egg production reduction). Emerging threats from porcine deltacoronavirus (PDCoV) (2021), novel canine coronavirus (2021), and HKU5-CoV-2 (2025) demonstrate ongoing zoonotic potential.

Accordingly, the development of broad-spectrum antivirals applicable across species is not only essential for the control of animal diseases but also a proactive strategy to forestall future outbreaks of novel human coronaviruses (Figure 2).

### 1.3. Advantages of Drug Repurposing

**Figure 3:**
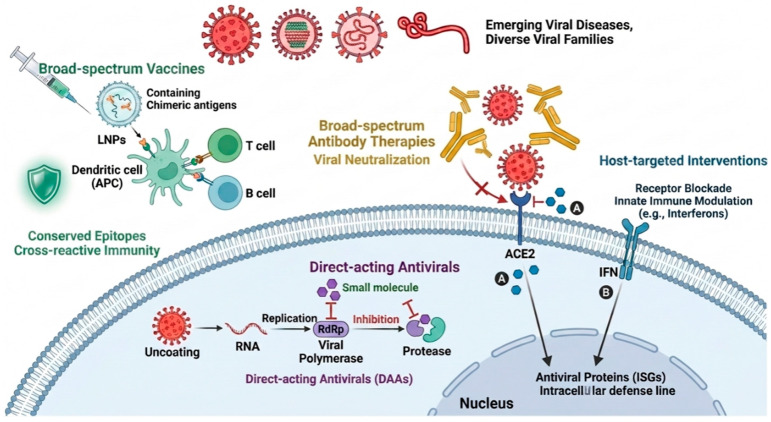
Overview of broad-spectrum antiviral strategies using coronaviruses as a representative example. Broad-spectrum vaccines induce cross-reactive immunity via conserved viral epitopes. Neutralizing antibodies block viral entry by inhibiting receptor binding. Direct-acting antivirals target viral enzymes such as RNA-dependent RNA polymerase (RdRp) to suppress replication. Host-directed therapies modulate host pathways to restrict infection.

To combat emerging and re-emerging coronavirus outbreaks, broad-spectrum antiviral strategies involve diverse technical approaches (Figure 3). Vaccine prevention represents the fundamental means for controlling epidemics [47,48]. However, confronted with the high-frequency mutation of coronaviruses (such as the successive emergence of SARS-CoV-2 Alpha, Delta, and Omicron variants) and their cross-species transmission characteristics, vaccine protective efficacy frequently wanes. Moreover, the cross-protective effects of animal coronavirus vaccines remain highly limited, and the timeline from research and development to large-scale administration still requires months to years. Monoclonal antibody therapy can provide immediate passive immunity, yet it remains highly susceptible to variant escape, entails substantial production costs, and its cold-chain storage and transportation requirements limit accessibility in resource-limited regions. Direct-acting antivirals (DAAs) target virus-specific enzymes (such as RdRp and 3CLpro) and possess well-defined antiviral mechanisms. Nevertheless, traditional de novo drug development comprises multiple successive stages, including target discovery, lead compound optimization, preclinical research, Phase I–III clinical trials and regulatory approval. This procedure typically takes 10–15 years, incurs substantial costs and suffers from very high failure rates, which hinders rapid clinical application within weeks or months amid sudden outbreaks [49]. Host-directed therapy (HDT) achieves antiviral effects by modulating conserved host pathways upon which viruses depend (such as metabolism, immunity, and receptors), theoretically possessing inherent broad-spectrum and anti-resistance advantages. However, the physiological complexity of host targets increases the risk of off-target toxicity, and long-term safety data remain scarce.

Among these strategies, drug repurposing offers a unique “fast track.” This strategy directly screens libraries of approved or clinically staged drugs, leveraging their existing pharmacokinetic, safety, and manufacturing supply chain data to bypass the most time-consuming early-stage development phases, thereby compressing the drug development cycle to 3–7 years and reducing costs by approximately 50–70% [50,51,52]. More importantly, drug repurposing inherently aligns with two core directions of broad-spectrum antiviral strategies: on one hand, by targeting highly conserved viral enzymes among coronaviruses (such as the Cys145–His41 catalytic dyad of 3CLpro and motif C of RdRp), it can achieve antiviral coverage across variants and genera; on the other hand, by intervening in conserved host factors upon which viruses depend (such as ACE2, TMPRSS2, and glycolysis/pyrimidine synthesis pathways), it can establish a “host defense line” insensitive to viral mutations, and due to the cross-species conservation of host pathways, it possesses translational potential for both human and veterinary applications.

Remdesivir represents a classic example of drug repurposing. Originally developed by Gilead Sciences as a broad-spectrum antiviral agent, it was subsequently investigated primarily for the Ebola virus in clinical studies [53]. Although its efficacy against the Ebola virus was limited, following the outbreak of COVID-19, researchers rapidly repositioned it as an anti-SARS-CoV-2 candidate drug based on its mechanism of inhibiting RdRp [54]. Leveraging previously accumulated preclinical and clinical safety data, remdesivir rapidly entered clinical trials for COVID-19 and received Emergency Use Authorization (EUA) from the U.S. Food and Drug Administration (FDA) in 2020 for the treatment of severe COVID-19 patients [55]. Major clinical trials demonstrated that it could shorten patient recovery time; however, its effect on reducing mortality was not significant, and its efficacy remains somewhat controversial. This process fully exemplifies the efficiency and practicality of the drug repurposing strategy in responding to emerging infectious diseases, while simultaneously revealing that its ultimate efficacy still requires thorough validation through rigorous clinical trials.

This review systematically summarizes advances in repurposed drugs that act on key coronavirus proteins and host factors from three perspectives: viral target conservation, host target homology, and cross-species immune modulation. Special focus is placed on their translational challenges for treating coronavirus infections in humans and animals.

## 2. Repurposed Drugs Regulating the Entry and Replication of Coronaviruses

### 2.1. Drugs Affecting Viral Adsorption and Entry

#### 2.1.1. Drugs Interfering with the Binding Between Viral Spike Protein and Host Receptors

The spike (S) protein is a key surface glycoprotein that mediates host cell entry of coronaviruses. The entry process driven by the S protein undergoes three core sequential steps: binding to specific host cell receptors, proteolytic priming by host proteases, and fusion between the viral envelope and cell membrane triggered by conformational changes [12,28,56]. Different coronaviruses utilize distinct host receptors. For instance, SARS-CoV and SARS-CoV-2 recognize angiotensin-converting enzyme 2 (ACE2), while MERS-CoV targets dipeptidyl peptidase 4 (DPP4). *Alphacoronaviruses* such as HCoV-229E and transmissible gastroenteritis virus (TGEV) bind to aminopeptidase N (APN/CD13) [57]. Repurposing strategies targeting the S protein mainly focus on blocking receptor binding and inhibiting proteolytic cleavage mediated by host proteases. Among these approaches, targeting the binding interface between viral particles and host receptors exhibits unique advantages. Unlike the S protein (especially its RBD), which has a high mutation rate, host receptors possess highly conserved structures. Their sequences and biological functions are rarely altered by viral mutations, and they share considerable homology across various species. This characteristic not only underpins the cross-species transmission capacity of certain coronaviruses but also endows such drugs with the potential for cross-species application and broad-spectrum antiviral activity.

Ursodeoxycholic acid (UDCA) is an endogenous bile acid widely used for the treatment of cholestatic liver diseases and gallstone dissolution. In recent years, studies have revealed its potential anti-coronavirus activity. It downregulates ACE2 expression in host cells by inhibiting the farnesoid X receptor (FXR), thereby reducing SARS-CoV-2 binding sites and blocking viral entry; Brevini et al. confirmed this effect in in vitro cell cultures, organoids, and animal models [58]. Clinical retrospective studies have also demonstrated that patients with liver diseases receiving long-term UDCA treatment exhibited significant reductions in hospitalization and severe illness rates following SARS-CoV-2 infection [59]. In the field of animal coronaviruses, UDCA can inhibit FIPV replication by activating the JAK-STAT signaling pathway to induce type I interferon production and can directly disrupt viral envelope structures to induce virion disintegration [60]; furthermore, in porcine respiratory coronavirus (PRCV) infection models, UDCA has also demonstrated in vivo antiviral activity, alleviating viral pneumonia-induced damage [61]. These mechanisms target cross-species conserved host pathways such as FXR-ACE2 and JAK-STAT, suggesting that UDCA possesses translational potential for the synergistic prevention and treatment of both human and animal coronavirus infections.

Lactoferrin (LF), a natural iron-binding glycoprotein, has been demonstrated to possess broad-spectrum antiviral activity against multiple human and animal coronaviruses. In human coronaviruses, its primary mechanism involves binding to HSPGs on the host cell surface, thereby blocking the initial attachment of the viral spike protein to HSPGs, reducing subsequent viral binding to ACE2 and other receptors, and ultimately inhibiting viral entry [26,62]. Furthermore, lactoferrin can directly bind to the RBD of the SARS-CoV-2 spike protein, competitively blocking viral binding to ACE2, and can inhibit the activity of viral RdRp, thereby interfering with viral replication [63,64]. In vitro studies have shown that bovine lactoferrin (bLF) exhibits inhibitory effects against SARS-CoV-2 and multiple human coronaviruses and demonstrates synergistic effects when combined with drugs such as remdesivir [26]. In the field of animal coronaviruses, porcine lactoferrin can similarly block the adsorption of PEDV spike protein to host cells through competitive binding to HSPG, significantly inhibiting viral replication; mechanistically, LF can also trigger dendritic cell maturation in piglets and enhance their antigen-presenting function, thereby improving intestinal mucosal immune responses [65]. Regarding clinical translation, lactoferrin has been proposed as an oral or intranasal supplement for the prevention and adjuvant treatment of COVID-19; however, large-scale clinical trial data are currently lacking [66,67].

Statins, as commonly used lipid-lowering drugs, have been extensively investigated in recent years for drug repurposing against human and animal coronavirus infections. Their potential anti-coronavirus mechanisms primarily include reducing membrane cholesterol levels and disrupting lipid raft structure via inhibition of HMG-CoA reductase, thereby altering the distribution and accessibility of ACE2 and other receptors on the cell membrane and interfering with early steps of viral entry; some studies further suggest that statins may downregulate CD147 expression, additionally affecting viral attachment and entry [68,69]. Statins can also modulate autophagic flux through pathways such as AMPK–mTOR–ULK1, promoting viral particle clearance or inhibiting viral replication in certain cellular and animal models [68]. Clinically, multiple prospective cohort studies and meta-analyses have demonstrated that prior or in-hospital statin use is significantly associated with lower risks of severe illness or mortality in COVID-19 patients (pooled HR approximately 0.65–0.73) [70,71]. Regarding animal coronaviruses, existing studies have shown that the infection processes of various animal coronaviruses (such as those in swine and feline species) depend on host cell membrane cholesterol or lipid raft structures; statins may theoretically inhibit coronavirus infection by disrupting cholesterol metabolism and lipid raft formation [72,73,74]. While this provides a mechanistic rationale for statin-mediated inhibition, empirical support from in vitro or in vivo studies has yet to be obtained.

Methyl-β-cyclodextrin (MβCD), a candidate agent for drug repurposing, primarily exerts anti-coronavirus activity by depleting cholesterol from cell membranes and disrupting lipid raft structures, which hinders the binding of coronaviruses to host cell receptors such as ACE2 and blocks viral entry [72,75]. In vitro studies have demonstrated that both MβCD and hydroxypropyl-β-cyclodextrin (HPβCD) can markedly inhibit the cell entry and replication of SARS-CoV-2 as well as HCoV-229E and other coronaviruses [75,76]. Animal experiments have further verified that β-cyclodextrin provides protection against SARS-CoV-2 infection in a hamster nasal model [75]. Similar to statins, MβCD interferes with cholesterol metabolism and thus holds potential to suppress animal coronaviruses. Additionally, MβCD can serve as a drug carrier to form inclusion complexes with antiviral agents such as oseltamivir and naphthoquinones, which enhances the anti-coronavirus potency and bioavailability of combined drugs [77]. Given its favorable safety profile and wide application in pharmaceutical formulations, MβCD and related cyclodextrin derivatives are regarded as promising candidates for clinical translation as broad-spectrum anti-coronavirus therapeutics. Nevertheless, large-scale clinical trial data are still lacking, and their efficacy and safety in humans require further validation.

Chloroquine (CQ) and hydroxychloroquine (HCQ) were originally approved for the treatment of malaria and autoimmune diseases. Early in vitro studies have demonstrated that CQ/HCQ can elevate the pH of endosomes and inhibit the pH-dependent conformational changes in the spike (S) protein, thereby blocking viral entry. Meanwhile, these compounds can raise the pH of the Golgi apparatus, interfere with the glycosylation of viral proteins, and suppress viral assembly and egress [78,79]. Molecular simulation studies suggest that CQ/HCQ can also impede viral attachment by inhibiting the binding between the S protein and sialic acids or gangliosides on the host cell surface [79,80]. In assays using Vero E6 cells with a multiplicity of infection (MOI) of 0.01, the half-maximal effective concentration (EC_50_) of HCQ against SARS-CoV-2 was 4.5–4.7 μM, slightly higher than that of CQ (EC_50_ ≈ 1.13 μM). However, HCQ achieves a much higher intracellular accumulation, reaching levels 50 to 100 times those in plasma, which endows it with superior overall antiviral activity [79]. Studies on FIPV and CCoV have shown that CQ can dramatically inhibit viral replication and the development of cytopathic effects, indicating its potential for the intervention of animal coronavirus infections [81,82]. Nonetheless, large-scale randomized controlled trials, including the RECOVERY trial (n = 15,420), failed to confirm clinical benefits of HCQ in hospitalized COVID-19 patients, with no reductions in mortality or disease progression observed [83,84]. Pharmacokinetic analyses indicated that achieving the plasma concentration (>1 μM) required for in vitro antiviral effects would demand doses far exceeding the safe range, which substantially increases the risk of cardiac toxicity such as QT interval prolongation and arrhythmias [85]. Based on these findings, leading international guidelines issued by the National Institutes of Health (NIH) and World Health Organization (WHO) no longer recommend CQ or HCQ for COVID-19 treatment.

Enoxaparin is a low molecular weight heparin (LMWH) primarily used for anticoagulant therapy in the treatment of COVID-19. Clinical studies have demonstrated that enoxaparin significantly reduces mortality among hospitalized patients while decreasing the need for intensive care and the risk of disease progression [86]. Beyond its anticoagulant effects, recent in vitro and mechanistic studies suggest that it may possess certain antiviral potential: enoxaparin can block viral entry by binding to the interface between the SARS-CoV-2 spike (S) protein and the ACE2 receptor; simultaneously, it can enhance the inhibitory effect of alpha-1-antitrypsin on TMPRSS2, thereby interfering with host protease-mediated cleavage (priming) of the S protein and viral entry [87,88]. In in vitro experiments, enoxaparin inhibited SARS-CoV-2 infection in Vero cells at concentrations ranging from 50 to 500 μg/mL, effectively reducing viral load and maintaining inhibitory activity in simulated human biological environments (such as sputum) [87]. Systematic data from animal coronavirus infection models are currently lacking; future studies should further validate their direct antiviral efficacy and potential for combination therapy in both human and animal coronavirus infections.

#### 2.1.2. Repurposed Drugs Targeting Host Proteases Involved in Viral Entry

The entry and replication processes of the vast majority of coronaviruses are highly dependent on viral glycoprotein processing mediated by specific host proteases. By inhibiting these host proteases, key steps required for viral replication can be interrupted, thereby blocking infection at its source. Among them, TMPRSS2 cleaves and activates the viral spike (S) protein at the cell surface, while cathepsin B/L facilitates S protein cleavage within the endosomal pathway. These two routes represent the major entry mechanisms utilized by coronaviruses [28,89]. Repurposed drugs targeting these host proteases can effectively inhibit viral entry, possessing the characteristic of ‘host-targeted, broad-spectrum inhibition’.

Camostat Mesylate: Approved in Japan in 2001 for the treatment of pancreatitis, camostat mesylate was rapidly advanced into drug repurposing research during the COVID-19 pandemic. As an oral serine protease inhibitor, its primary anti-coronavirus mechanism involves the inhibition of the TMPRSS2 protease on the host cell surface, thereby blocking the activation of the spike (S) proteins of SARS-CoV-2 and other coronaviruses and preventing ACE2 receptor-mediated cell membrane fusion and viral entry [90,91]. Molecular modeling and structural studies have demonstrated that camostat forms stable interactions with key residues within the TMPRSS2 active site (such as His296 and Ser441), exerting potent inhibitory effects [92,93]. In vitro experiments have shown that both camostat and GBPA can effectively inhibit infection by SARS-CoV, SARS-CoV-2, and MERS-CoV in Calu-3 and other human lung epithelial cells [28,94]. Regarding clinical investigations, Phase I trials have confirmed that high-dose oral camostat is safe and well-tolerated, while early open-label trials indicated improvements in organ function and inflammatory markers in some patients with severe COVID-19; however, multiple randomized controlled trials have not identified significant improvements in viral clearance rates or mortality [90,95,96]. Currently, no experimental data have demonstrated that camostat can effectively inhibit animal coronaviruses; nevertheless, given the conserved role of TMPRSS2 in the entry of various coronaviruses, camostat holds potential for cross-species applications [97,98].

Amantadine is a classic ion channel blocker initially developed in the 1960s for the prevention and treatment of influenza A virus infection and has been widely used in Parkinson’s disease therapy for over five decades. In recent years, following the outbreak of SARS-CoV-2, researchers have identified its potential antiviral activity against coronaviruses. Existing studies indicate that amantadine exhibits certain antiviral activity against SARS-CoV-2 in vitro, with mechanisms including the downregulation of host cell lysosome-related genes (such as Cathepsin L), thereby interfering with viral spike protein cleavage and the viral entry process, as well as the inhibition of current activity of SARS-CoV-2-encoded ion channel proteins (such as the E protein and ORF10), suggesting its potential as a viroporin inhibitor affecting viral assembly and release [99,100]. In vitro experiments have shown that amantadine significantly inhibits SARS-CoV-2; however, the IC_50_ concentration for SARS-CoV-2 exceeds levels achievable with conventional clinical dosing, suggesting that its in vivo antiviral efficacy may be limited unless local high-concentration administration routes are employed [101,102]. In summary, amantadine can inhibit SARS-CoV-2 through multi-target mechanisms in vitro, but high-quality animal experimental and clinical data are currently lacking, and studies on its antiviral effects in animal coronavirus infection models are absent.

E-64d, as a cysteine protease inhibitor, has attracted attention in drug repurposing research against both human and animal coronaviruses. Its core mechanism involves the inhibition of host cell cysteine proteases such as Cathepsin L, thereby blocking the endosomal escape process mediated by this enzyme in viruses such as SARS-CoV-2 and MHV and suppressing viral genome release and replication [103,104]. Additionally, E-64d and certain analogous inhibitors can act on the active center of the coronavirus 3CLpro, inhibiting polyprotein cleavage and further interfering with viral replication [105]. In vitro studies have shown that E-64d can significantly inhibit SARS-CoV-2 pseudovirus infection and dose-dependently suppress the replication and RNA synthesis of the animal coronavirus MHV in cells [103,104]. Notably, the combination of E-64d and TMPRSS2 inhibitors (e.g., camostat mesylate) enables dual-pathway blockade of coronavirus entry and produces synergistic antiviral effects in SARS-CoV-2 infection models [106]. Currently, research on E-64d and related compounds against coronaviruses remains primarily at the stages of molecular mechanism, in vitro cellular, and animal cell experiments, with no human clinical trial data available; thus, their clinical translational value still requires further validation through animal studies and clinical investigations.

Nafamostat mesylate is a serine protease inhibitor approved in Japan, China, and other regions, with nafamostat mesylate being the primary form used in clinical practice, mainly for anticoagulation during blood purification therapy. In recent years, it has garnered extensive attention in drug repurposing research for coronavirus infections. Its core mechanism involves the potent inhibition of the TMPRSS2 protease on the host cell surface, thereby blocking spike protein-mediated membrane fusion and cell entry by SARS-CoV-2, MERS-CoV, and other coronaviruses, and significantly suppressing viral infection [107,108,109]. In vitro experiments have demonstrated that nafamostat exhibits far greater inhibitory potency against SARS-CoV-2 than the analogous drug camostat, with effective concentrations falling below routine clinical plasma levels [107,109]. In animal experiments, intranasal administration of nafamostat significantly reduced pulmonary viral load, body weight loss, and mortality in human ACE2 transgenic mice, demonstrating favorable in vivo antiviral activity [110]. Okugawa et al. conducted a multicenter randomized controlled trial and found that nafamostat mesylate exerted antiviral effects in patients with mild, early-stage COVID-19, with generally favorable safety; however, some patients experienced adverse reactions such as phlebitis [111]. Overall, nafamostat demonstrates anti-coronavirus potential through multi-target mechanisms, including TMPRSS2 inhibition, as evidenced by in vitro studies, animal models, and early clinical research; however, its clinical translational value in animal coronavirus infections and broader populations requires validation through additional high-quality clinical trials.

### 2.2. Drugs Affecting Viral Replication

#### 2.2.1. Agents Targeting RNA-Dependent RNA Polymerase

In the life cycle of coronaviruses, RdRp is the core enzyme complex governing viral replication and transcription. This complex is composed of the catalytic subunit nsp12, along with its cofactors nsp7 and two nsp8 molecules, and it mediates the synthesis of genomic RNA and subgenomic mRNA [23,112]. The palm domain of nsp12 harbors a highly conserved motif C, which typically contains the SDD sequence and acts as a vital target for broad-spectrum antiviral drugs [23]. Nucleoside analogs exert pharmacological effects through two primary mechanisms: competitive incorporation followed by chain termination or the induction of error catastrophe. They are the main class of repurposed drugs developed against this target [113,114,115].

Remdesivir was originally developed for the Ebola virus and was repositioned in 2020 to become the first FDA-approved antiviral drug for COVID-19. In vivo, it is metabolized to its triphosphate form (GS-443902), which competes with natural ATP and is incorporated by viral RdRp into nascent RNA chains, causing delayed chain termination and thereby blocking synthesis of viral genomic and subgenomic RNA [113,116]. As a nucleotide analogue prodrug, remdesivir’s clinical efficacy remains highly controversial. The NIH-led ACTT-1 randomized controlled trial demonstrated that it could shorten recovery time in hospitalized patients (median 11 days vs. 15 days) [55]; however, the WHO “Solidarity Trial” multicenter study found that it did not significantly reduce mortality [117]. Subsequent studies suggest that remdesivir has limited efficacy in severe or late-stage COVID-19 patients [118], indicating that its clinical benefit may depend on patient population and disease stage, requiring individualized use. The major metabolite of remdesivir, GS-441524, has been widely used against animal coronavirus infections, particularly feline infectious peritonitis (FIP). In vitro experiments show that its EC_50_ against FIPV is approximately 0.78 μM, with no significant toxicity to feline cells [119]. Clinical and retrospective studies indicate that the overall cure rate of GS-441524 is 77–96%, and the cure rate for neurological FIP can exceed 80% through high-dose regimens (10–15 mg/kg/day) [119,120]. In the field of swine coronaviruses, remdesivir and its metabolite GS-441524 exhibit potent in vitro inhibitory activity against PEDV [121,122]. Moreover, ATV014, a novel orally available broad-spectrum anti-coronavirus derivative of GS-441524, displays over 2.5-fold, 8-fold and 12-fold enhanced antiviral potency relative to GS-441524 against PEDV, SADS-CoV and TGEV, with respective EC_50_ values of 0.294, 0.414 and 0.629 μM [123]. However, GS-441524 remains in a state of being “scientifically effective yet commercially and legally stalled,” facing obstacles in clinical translation.

Molnupiravir is an oral nucleoside analogue prodrug approved for COVID-19 treatment in 2021. In vivo, it is metabolized to the active form β-D-N4-hydroxycytidine triphosphate (NHC-TP), which can be erroneously incorporated by viral RdRp into nascent chains, inducing the accumulation of G→A and C→U mutations and thereby causing “lethal mutagenesis” that inactivates the viral population [115]. In studies on human coronaviruses, therapeutically administered ribonucleoside analogue Molnupiravir (MK-4482/EIDD-2801) has been proven to block SARS-CoV-2 transmission in ferret models [124]. The Phase III MOVe-OUT clinical trial demonstrated that molnupiravir reduced the risk of hospitalization or death within 29 days from 9.7% to 6.8% (a relative reduction of 30%) in mild-to-moderate COVID-19 patients with high-risk factors [125]. However, the 2023 Lancet PANORAMIC large-scale randomized controlled trial suggested that its overall clinical benefit is limited [126]. Regarding animal coronaviruses, molnupiravir dose-dependently inhibits the replication of PEDV and various *alphacoronaviruses* (including FCoV, CCoV and TGEV) in vitro and can induce mutation accumulation [127,128]. Overall, molnupiravir has achieved clinical application in human COVID-19 treatment but remains primarily at the experimental stage in the field of animal coronaviruses.

Azvudine (FNC) is a synthetic nucleoside analogue originally developed as a reverse transcriptase inhibitor for HIV infection. In HIV research, FNC is phosphorylated intracellularly to its active triphosphate form (FNC-TP), which can inhibit reverse transcriptase activity and, to a certain extent, counteract the Vif protein-mediated degradation of the host restriction factor APOBEC3G, thereby interfering with viral replication [129]. Regarding coronaviruses, FNC-TP can be incorporated by RdRp into nascent RNA chains, blocking viral RNA synthesis and inhibiting replication. In vitro experiments indicate that the half-maximal effective concentration (EC_50_) of azvudine against SARS-CoV-2 is approximately 1.2–4.3 μM, and it also exhibits inhibitory effects against the *Betacoronavirus* HCoV-OC43; moreover, it demonstrates a high selectivity index [130]. In animal studies, experiments in rats and rhesus macaques revealed that the active metabolite of azvudine accumulates markedly in the thymus and peripheral blood mononuclear cells, exhibiting “thymus tropism”. This metabolite can lower SARS-CoV-2 viral loads across multiple tissues, mitigate pulmonary pathological lesions and improve immune dysregulation [130]. Subsequently, several prospective cohort studies, real-world retrospective studies, and systematic reviews have suggested that azvudine can shorten the time to viral nucleic acid negativity, accelerate symptom recovery, and reduce case fatality rates, with generally favorable safety profiles [131,132]. However, these clinical data are largely derived from observational or limited-sample studies with heterogeneous results, and high-quality, large-scale randomized controlled trials remain ongoing.

Suramin is a classic repurposed drug with a century-long history of clinical use, originally approved for the treatment of African trypanosomiasis and onchocerciasis, and was subsequently identified as having inhibitory activity against SARS-CoV-2 through drug repurposing screens. Its antiviral action relies on a multi-target synergistic mechanism: in the viral replication stage, suramin can specifically bind to the template strand-binding site and catalytic active center of SARS-CoV-2 RdRp, competitively blocking the interaction between nucleic acids and the enzyme through its strong negative charge, thereby directly inhibiting RdRp catalytic activity; enzymatic experiments have confirmed that its inhibitory effect is significantly superior to the active form of remdesivir [133]; Apart from RdRp, suramin also displays antiviral effects against SARS-CoV-2 by acting on the S protein and N protein at distinct stages of the viral life cycle [134,135]. Suramin has clearly demonstrated anti-SARS-CoV-2 activity at low micromolar concentrations in cell models such as Vero E6, effectively reducing viral load and alleviating cytopathic effects [133,134]. Currently, although the in vitro antiviral mechanism of suramin has been clearly elucidated via structural biology and related investigations, in vivo pharmacodynamic and safety assessments remain limited.

#### 2.2.2. Targets 3c-like Protease and Papain-like Protease

3CLpro/Mpro is an essential enzyme for the replication of human and animal coronaviruses. It cleaves viral polyproteins pp1a/pp1ab to produce multiple non-structural proteins that are required for viral replication and transcription. Its canonical cleavage site is Leu-Gln↓(Ser, Ala, Gly), and the active center is composed of a catalytic dyad of Cys145 and His41. These structural characteristics are highly conserved across α-, β- and γ-coronaviruses, and no homologous protease exists in host cells [136,137]. For this reason, 3CLpro is regarded as a high-selectivity and low-toxicity antiviral target applicable to both human and animal coronaviruses. Meanwhile, PLpro exerts dual functions: it not only cleaves viral polyproteins to accomplish protein processing for viral replication but also suppresses host innate immunity, especially the interferon signaling pathway, via deubiquitination and deISGylation, thus helping viruses evade host immune surveillance [138,139,140]. Consequently, inhibiting PLpro can simultaneously block viral replication and restore or strengthen host antiviral immunity, rendering it a promising drug target with both direct and indirect antiviral effects. In terms of drug repurposing, numerous natural products and conventional drugs have been confirmed to inhibit 3CLpro and PLpro, which opens up an important avenue for developing broad-spectrum anti-coronavirus agents.

GC373 is the parent compound of GC376. Both compounds were initially developed to target the 3CL protease of FCoV, and GC376 has been clinically applied for the treatment of feline infectious peritonitis (FIP). Subsequent studies have found that GC373 and its prodrug GC376 can effectively inhibit the 3CL protease of human coronaviruses, including SARS-CoV and SARS-CoV-2 with nanomolar IC_50_ values, and markedly suppress viral replication in cell cultures [141,142]. Mechanistically, they form reversible hemithioacetal linkages to covalently bind to Cys145, a key residue within the active site of the 3CL protease. The binding complex is further stabilized at the active site via hydrophobic interactions and hydrogen bond networks, which block the cleavage of viral polyproteins and inhibit viral replication [141,143]. To date, GC373 and GC376 remain in the experimental stage for the treatment of human coronavirus infections and have not been introduced into clinical use. However, results from cellular and animal studies demonstrate their broad-spectrum anti-coronavirus potential.

Baicalein, one of the major active ingredients of Shuanghuanglian oral liquid, has garnered extensive attention in research on drug repurposing against coronavirus infections in recent years. Studies have verified that baicalein exerts potent inhibitory effects on SARS-CoV-2 3CLpro, with an IC_50_ of 0.39 μmol/L. Its EC_50_ against viral replication in Vero E6 cells is 2.94 μmol/L, indicating low cellular cytotoxicity and a broad safety window [144]. In addition to human coronaviruses such as SARS-CoV-2 and SARS-CoV, baicalein can suppress the 3CLpro activity and replication of multiple animal coronaviruses, including PEDV, FIPV, bovine coronavirus (Bovine-CoV) and HCoV-OC43, thus exhibiting remarkable broad-spectrum antiviral activity [145,146]. Crystal structure analyses reveal that baicalein binds to the active site of 3CLpro as a non-covalent and non-peptidomimetic small molecule. It uniquely inserts between the two catalytic residues His41 and Cys145 and functions as a molecular shield to block substrate entry into the catalytic center. This mechanism prevents the proteolytic cleavage of viral polyproteins and ultimately suppresses viral replication [147,148]. Featuring this distinctive binding mode and high ligand binding efficiency attributed to its compact molecular scaffold, baicalein serves as a valuable lead compound for the further development of 3CLpro inhibitors. It is also among the rare non-covalent and non-peptidomimetic small-molecule inhibitors discovered so far.

Boceprevir, a clinically approved drug for hepatitis C virus (HCV) infection, has been proven to inhibit coronavirus replication by targeting the 3CLpro of SARS-CoV-2. It exerts potent anti-SARS-CoV-2 activity and effectively suppresses viral replication in Vero cells [142]. Crystal structure analysis demonstrates that boceprevir primarily binds to the catalytic active site of 3CLpro, thereby blocking the cleavage and maturation of viral polypeptides [142,149]. Further in vitro and cellular experiments reveal that boceprevir possesses broad-spectrum antiviral activity against SARS-CoV-2 and other coronaviruses, including SARS-CoV, MERS-CoV and multiple human coronaviruses [150]. Nevertheless, studies have found that long-term administration of boceprevir may induce mutations at the 3CLpro site, leading to drug resistance to this compound and related inhibitors such as nirmatrelvir [151]. Therefore, although boceprevir, as a 3CLpro inhibitor, provides important insights for the development of anti-SARS-CoV-2 therapeutics, its drug resistance issues and clinical application prospects still require further investigation.

Chlorogenic acid is one of the major active constituents of plants such as honeysuckle, and it was approved as an animal feed additive in China in 2019. Molecular docking and simulation studies have demonstrated that chlorogenic acid has high binding affinity for both the 3CLpro and ACE2 receptors of SARS-CoV-2, suggesting that it may exert antiviral effects by inhibiting 3CL protease activity and blocking the interaction between viral spike (S) protein and ACE2 [152]. In addition, chlorogenic acid modulates signaling pathways such as MDA5, TLR7 and NF-κB, upregulates the expression of interferons and immunoglobulins, and enhances anti-inflammatory and immunomodulatory functions [153]. In in vitro assays, chlorogenic acid exerts prominent inhibitory effects against animal coronaviruses, including PDCoV and IBV; meanwhile, it reduces virus-induced apoptosis and inflammatory responses [153,154,155]. At present, relevant research is mainly confined to in vitro assays and animal models. Further preclinical pharmacodynamic evaluation and safety verification are needed to assess its application potential as a broad-spectrum anti-coronavirus agent.

Carmofur, as an antitumor agent, has been demonstrated to inhibit the activity of the coronavirus 3CLpro through covalent binding to the catalytic site Cys145, thereby blocking cleavage of the viral polyprotein and viral replication [156]. X-ray crystal structures reveal that the carbonyl active group of carmofur forms a covalent bond with Cys145, while its fatty acid tail occupies the hydrophobic S2 subsite of 3CLpro [156]. In vitro experiments indicate that the EC_50_ of carmofur against SARS-CoV-2 is approximately 24.3 μM, and it exhibits a similar binding mode against MERS-CoV, suggesting its potential broad-spectrum anti-coronavirus activity [156,157]. Structure-optimized derivatives based on carmofur have demonstrated stronger 3CLpro inhibitory activity against SARS-CoV-2 in vitro (IC_50_ as low as 0.35 μM), yet their antiviral activity (EC_50_) remains in the 20–30 μM range, indicating that therapeutic efficacy needs improvement [158]. Furthermore, some studies have noted that carmofur and analogous molecules exhibit limited inhibitory effects in cell models, with decreased activity under reducing conditions that more closely resemble the in vivo environment [158,159]. Currently, no data from animal models or clinical anti-coronavirus studies are available for carmofur, and its clinical translation toward coronavirus infection treatment still requires further in vivo pharmacodynamic and safety assessment.

Disulfiram, as a classic alcohol-aversion drug, has demonstrated potential value in drug repurposing research for coronavirus infections. In vitro studies have shown that disulfiram can inhibit the 3CLpro of multiple coronaviruses; its mechanism of action involves covalent modification of key cysteine residues, thereby blocking cleavage of the viral polyprotein and inhibiting viral replication [160]. Notably, disulfiram is effective not only against SARS-CoV-2 3CLpro but can also inhibit the 3CLpro of various coronaviruses including MERS-CoV and PEDV, suggesting certain broad-spectrum anti-coronavirus potential [160]. Furthermore, disulfiram can target multiple conserved zinc-binding sites in the viral RTC; when combined with drugs such as remdesivir, it produces synergistic inhibitory effects, improving antiviral efficiency and reducing the risk of drug resistance [161]. Clinically, retrospective cohort studies based on the US Veterans database have shown that disulfiram users had approximately a 34% lower risk of SARS-CoV-2 infection compared with the control group, and no deaths were observed among infected individuals, suggesting that it may reduce COVID-19 infection rates and disease severity [162]. However, no formally published randomized controlled trial results are currently available; existing evidence derives primarily from in vitro experiments and retrospective epidemiological analyses.

Ebselen is an organoselenium compound that has been clinically evaluated as an antioxidant/cytoprotective agent in human studies and has been repeatedly reported as a multi-target antiviral candidate in drug repurposing research for coronaviruses. In vitro and structural biology evidence indicates that ebselen exerts inhibitory effects against both the 3CLpro and PLpro of SARS-CoV-2; crystallographic and mass spectrometric analyses reveal that it can undergo selenium–sulfur chemical reactions (selenation/selenosulfide formation) with catalytic cysteine residues of these enzymes, thereby interfering with enzymatic activity and blocking viral polyprotein cleavage [163,164]. Ebselen also acts as a “zinc-ejector” molecule, targeting zinc-containing viral replication complexes (such as the zinc-finger domains of nsp13/nsp14), inhibiting associated enzymatic activities, and producing synergistic effects with nucleoside analogues (such as remdesivir), thereby providing strategic evidence for a “multi-target + combination therapy” approach [161]. At the cellular/virological level, ebselen and its derivatives can inhibit SARS-CoV-2 replication at low micromolar concentrations (EC_50_ values mostly in the low micromolar range), with inhibition of 3CLpro and PLpro validated through chemical and cytological methods [161,163,164,165]. However, most current anti-coronavirus evidence for ebselen remains limited to in vitro enzymology, cellular infection, and structural studies, lacking systematic animal infection models and randomized controlled clinical trial data.

#### 2.2.3. Drugs Indirectly Affecting Viral Replication

In addition to directly targeting core enzymes involved in viral replication (such as RdRp, 3CLpro, and PLpro), another class of drugs intervenes in the coronavirus replication cycle through indirect mechanisms. Rather than acting directly on the enzymatic active centers encoded by the virus, these strategies exploit the inherent physiological processes of host cells to erect multiple “barriers” against viral replication. Because the host pathways involved are highly conserved across diverse coronaviruses and even broader viral families, such interventions often possess an inherently broad-spectrum nature and impose lower selective pressure for drug resistance. More importantly, modulating the host cellular environment can simultaneously exert inhibitory effects at multiple stages of the viral life cycle—such as entry, uncoating, genome release, protein translation, and assembly—thereby forming a multi-layered antiviral defense.

Nitazoxanide (NTZ) was initially approved in the United States in 2002 for the treatment of parasitic infections and was subsequently discovered to possess broad-spectrum antiviral activity. Its antiviral mechanism remains incompletely elucidated; existing studies suggest that NTZ exerts effects through multi-target actions: on one hand, it can enhance host innate immune responses (such as activating the RIG-I/IFN signaling pathway), and on the other hand, it affects viral protein maturation and processing at post-entry stages of the viral life cycle (including N-glycosylation modification of the spike protein), thereby inhibiting viral protein expression and replication [166,167,168]. Regarding coronavirus research, in vitro experiments have shown that NTZ exhibits inhibitory effects against multiple human coronaviruses (such as HCoV-229E, HCoV-NL63, HCoV-OC43, SARS-CoV-2, and MERS-CoV), reducing viral RNA levels and blocking viral replication [169,170]. A randomized controlled trial published in the European Respiratory Journal demonstrated that early application of NTZ could significantly reduce viral load in mild COVID-19 patients but did not significantly improve clinical outcomes [171]. In the field of animal coronaviruses, direct investigations of nitazoxanide remain currently absent. Future research should prioritize the evaluation of its antiviral activity in infection models such as PEDV and TGEV so as to clarify its practical translational potential from human coronaviruses to veterinary applications.

Niclosamide, an antiparasitic agent, has demonstrated broad potential against both human and animal coronaviruses in recent years. In human coronaviruses, it acts through host-directed mechanisms: impairing cellular pathways required for viral entry, interfering with viral internalization, and disrupting endosomal acidification to inhibit viral replication [172,173]. In vitro studies show that niclosamide exerts potent inhibitory effects against SARS-CoV and SARS-CoV-2, with IC_50_ values in the submicromolar range [173,174]. In the field of animal coronaviruses, niclosamide’s antiviral potential has been directly validated. Wang et al. (2023) screened an FDA-approved drug library using recombinant PEDV and identified niclosamide as exhibiting the strongest antiviral activity against PEDV, with an EC_50_ of 0.246 μmol/L and a selectivity index of 102.8 [175]. Mechanistic studies revealed that niclosamide dose-dependently inhibits viral RNA synthesis and protein expression of both classical and variant PEDV strains, primarily by blocking viral internalization rather than viral attachment [175]. In animal models, niclosamide-lysozyme composite inhalation formulations can be effectively delivered to the respiratory tract, significantly inhibiting SARS-CoV-2 and MERS-CoV infection while alleviating pulmonary inflammation [176].

## 3. Repurposed Drugs Modulating Host Pathological Pathways

### 3.1. Drugs Modulating Host Immune and Inflammatory Pathways

Severe coronavirus infections are frequently complicated by a “cytokine storm,” characterized by aberrant elevation of multiple pro-inflammatory cytokines—including interleukin-6 (IL-6), tumor necrosis factor-α (TNF-α), and interferon-γ (IFN-γ)—resulting in acute respiratory distress syndrome (ARDS), multiple organ injury, and high mortality [177,178,179]. In response to the cytokine storm, agents that modulate host immune and inflammatory pathways are hypothesized to improve clinical outcomes by attenuating hyperinflammation and mitigating tissue damage. Given the high conservation of these immune pathways across mammals, such drugs are theoretically amenable to cross-species application.

Tocilizumab is a humanized anti-interleukin-6 (IL-6) receptor monoclonal antibody approved for the treatment of rheumatoid arthritis. Its mechanism of action in coronavirus infections primarily involves blocking the binding of IL-6 to its receptor, thereby suppressing inflammatory signaling pathways and attenuating the cytokine storm and associated tissue damage [180,181]. Multiple clinical studies and systematic reviews have demonstrated that tocilizumab significantly reduces inflammatory markers in patients with severe coronavirus infections (particularly COVID-19), decreases the requirement for mechanical ventilation, shortens hospitalization duration, and lowers mortality in certain studies [180,182]. However, results from some high-quality randomized controlled trials and systematic reviews have shown heterogeneity, with certain studies failing to identify significant reductions in mortality or improvements in primary clinical outcomes [183,184]. In the field of animal coronaviruses, direct studies on tocilizumab remain completely absent. Beyond the lack of scientific evidence, its high production costs as a biologic agent and intravenous administration route also severely constrain its scalable application in livestock and poultry production.

Anakinra is a recombinant human interleukin-1 receptor antagonist (IL-1Ra) that blocks the activity of pro-inflammatory cytokines IL-1α and IL-1β and is indicated for the treatment of autoinflammatory diseases [185]. SARS-CoV-2 infection can activate the NLRP3 inflammasome and induce excessive IL-1β release; consequently, anakinra is regarded as an important host-directed therapeutic agent for controlling inflammatory responses in severe COVID-19 [179,186]. Multiple clinical studies, including the phase III SAVE-MORE randomized controlled trial, have shown that early administration of anakinra to patients at high inflammatory risk (e.g., soluble urokinase plasminogen activator receptor ≥ 6 ng/mL) significantly reduces progression to severe disease and 28-day mortality, improves oxygenation indices, and shortens hospitalization duration [187,188]. Systematic reviews and meta-analyses have further confirmed that anakinra exhibits definitive efficacy in reducing the rates of severe illness and mortality in COVID-19, with a relatively low risk of adverse events such as secondary infections [189]. Collectively, by targeting host inflammatory pathways rather than acting through direct antiviral mechanisms, anakinra offers a safe and effective immunomodulatory therapeutic strategy for severe coronavirus infections.

Fluvoxamine, a selective serotonin reuptake inhibitor (SSRI) for obsessive–compulsive disorder and depression, exerts anti-coronavirus effects through three core mechanisms. (1) Sigma-1 receptor agonism: Among SSRIs, fluvoxamine has the highest sigma-1 receptor affinity and agonistic activity [190,191]. Since early SARS-CoV-2 replication relies on sigma-1 receptors, receptor knockdown markedly suppresses viral replication [191]. It alleviates cytokine storm and systemic inflammation by regulating endoplasmic reticulum stress [192]. (2) Acid sphingomyelinase (ASM) inhibition: As a functional inhibitor of acid sphingomyelinase (FIASMA), fluvoxamine blocks the SARS-CoV-2-triggered ASM/ceramide pathway, prevents ceramide-rich membrane domain formation, and further inhibits ACE2 aggregation, viral endocytosis and endolysosomal transport [193,194]. (3) Immune and coagulation modulation: It inhibits platelet aggregation and mast cell degranulation, cuts IL-6 and other proinflammatory cytokines, and ameliorates hypercoagulability. Clinically, Lenze et al.’s randomized controlled trial first proved fluvoxamine reduces deterioration risk in ambulatory COVID-19 patients [195]. Large randomized platform trials verified its ability to lower emergency admission or hospitalization risks for high-risk outpatients [196]. Recent meta-analyses and systematic reviews also show daily fluvoxamine ≥200 mg cuts clinical worsening and hospitalization rates with favorable safety [197]. Still, several trials found no significant benefits, implying efficacy depends on baseline patient risk, dosage and treatment timing [198,199]. Collectively, existing data support fluvoxamine’s early protective effects against COVID-19 via anti-inflammation and immunoregulation, yet no relevant studies or translational evidence are available for animal coronavirus treatment.

Baricitinib is an oral Janus kinase (JAK1/JAK2) inhibitor originally developed for the treatment of rheumatoid arthritis. Its anti-coronavirus mechanism is characterized by a dual mode of action: on one hand, it suppresses the JAK-STAT signaling pathway, thereby reducing the production of multiple pro-inflammatory cytokines (such as IL-6, TNF-α, and GM-CSF) and alleviating the cytokine storm and tissue inflammatory damage; on the other hand, it inhibits Numb-associated kinases including AAK1 and GAK, interfering with the fusion of the viral envelope with the host cell membrane and blocking the ACE2-mediated endocytic entry pathway of SARS-CoV-2, thereby reducing viral replication and dissemination [200,201]. Multiple randomized controlled trials and systematic reviews have demonstrated that baricitinib in combination with standard-of-care therapy (such as dexamethasone or remdesivir) significantly reduces mortality, progression to severe disease, and hospitalization duration in hospitalized COVID-19 patients; its anti-inflammatory effects contribute to controlling the cytokine storm in severe patients, improving oxygenation indices, and decreasing the requirement for mechanical ventilation [202,203,204]. However, some studies have also indicated that the immunosuppressive effects of baricitinib may delay viral clearance and increase the risk of secondary infections (e.g., bacterial pneumonia, herpes zoster, and fungal infections); therefore, a thorough assessment of the patient’s immune status and infection risk is warranted prior to administration [203,205]. Although the JAK-STAT signaling pathway plays a similarly critical role in the host inflammatory response to various animal coronaviruses, experimental data validating the antiviral or anti-inflammatory efficacy of baricitinib in such infections remain scarce.

Dexamethasone is a classic glucocorticoid that, owing to its potent anti-inflammatory and immunomodulatory properties, was demonstrated during the COVID-19 pandemic to significantly reduce mortality in severe COVID-19 patients, particularly those requiring supplemental oxygen or mechanical ventilation. Its mechanisms primarily involve suppressing excessive immune responses and the cytokine storm, thereby mitigating ARDS and pulmonary injury [206,207]. Molecular and cellular studies have revealed that dexamethasone modulates neutrophil subsets, downregulates interferon-stimulated gene expression, and promotes the expansion of immunosuppressive immature neutrophils, thereby reshaping the inflammatory microenvironment [208]. Large-scale randomized controlled clinical trials (such as the RECOVERY trial) and multicenter studies have confirmed that dexamethasone significantly reduces 28-day mortality and the need for mechanical ventilation in severe COVID-19 patients [206,207]. Recent studies have further elucidated that dexamethasone exerts its life-saving anti-inflammatory effects by reversing the immunodysregulated state of monocytes in severe COVID-19 patients and restoring their normal inflammatory regulatory functions [209]. Direct in vivo studies in animal coronaviruses have been reported: in porcine respiratory coronavirus (PRCV) infection models, dexamethasone reduces pulmonary Th1 cytokines and attenuates lung injury, yet also suppresses antiviral immunity and increases viral load [210]; in infectious bronchitis virus (IBV) chicken models, dexamethasone induces immunosuppression and upregulates cholesterol metabolism to promote viral replication [211]. These findings suggest a double-edged sword effect of glucocorticoids in animal coronavirus infections—where anti-inflammatory benefits must be weighed against the risks of immunosuppression.

Cetaben is a classic acyl-coenzyme A:cholesterol acyltransferase (ACAT, also known as SOAT) inhibitor originally developed as a cholesterol-lowering drug. ACAT catalyzes the esterification of free cholesterol to cholesterol esters, playing a central role in maintaining cellular cholesterol homeostasis, membrane fluidity, and lipid raft formation [212]. The life cycle of coronaviruses is highly dependent on host cell membrane cholesterol: viruses bind to host receptors through cholesterol-enriched lipid raft regions (such as GM1 microdomains) and complete endocytic invasion, while the formation of viral replication complexes also requires membrane cholesterol [213,214]. In 2025, the team led by Yongtao Li at Henan Agricultural University screened 117 cholesterol-lowering compounds and discovered for the first time that cetaben significantly inhibits PEDV replication [215]. Regarding SARS-CoV-2, the direct antiviral effect of cetaben has not yet been experimentally validated; however, the ACAT inhibitor Avasimibe, which belongs to the same class, has been demonstrated to inhibit SARS-CoV-2 entry and replication in human primary bronchial epithelial cells, suggesting that cetaben may possess similar broad-spectrum cross-species activity [216].

### 3.2. Drugs Targeting Host Metabolic Pathways

Coronavirus replication is dependent upon host cellular metabolic resources, as these pathways provide the energy and raw materials necessary to support viral replication and assembly [217,218,219]. Drugs targeting host metabolic pathways can effectively inhibit viral replication and spread by “depriving the virus of nutrients required for replication” or interfering with critical metabolic steps. Such metabolic modulation strategies possess inherent broad-spectrum antiviral potential; moreover, given the high conservation of metabolic pathways across mammals, they are theoretically amenable to cross-species application.

Leflunomide is an approved isoxazole-class drug with anti-rheumatic and immunosuppressive properties. Its primary mechanism of action is the inhibition of dihydroorotate dehydrogenase (DHODH), which blocks de novo pyrimidine synthesis and exerts antiproliferative effects on T and B lymphocytes. Meanwhile, leflunomide reduces the levels of proinflammatory cytokines such as IL-6 and TNF-α via suppression of the JAK/STAT and NF-κB signaling pathways, thereby producing immunomodulatory and anti-inflammatory effects [220,221,222]. Leflunomide confers antiviral activity against a variety of coronaviruses, including human coronaviruses (SARS-CoV-2, HCoV-229E, HCoV-OC43) and animal coronaviruses exemplified by PEDV [222,223,224]. In animal experiments, treatment with DHODH inhibitors markedly decreases viral loads and alleviates intestinal lesions in PEDV-infected pig models, verifying the feasibility of metabolism-targeted strategies for the management of animal coronavirus infections [222]. Multiple clinical studies have verified the moderate therapeutic efficacy of leflunomide in COVID-19 patients. Randomized controlled trials focusing on Omicron variant infection have confirmed its capacity to shorten fever duration and the time to viral RNA clearance [225,226]. In summary, owing to its dual mechanisms of metabolic inhibition and immunomodulation, leflunomide possesses therapeutic potential against both human and animal coronaviruses.

Pralatrexate, a dihydrofolate reductase (DHFR) inhibitor approved by the U.S. FDA, has garnered attention in recent years for drug repurposing against human coronaviruses. Its antiviral mechanism primarily involves the inhibition of folate metabolism in host cells, reducing nucleotide synthesis and thereby creating a shortage of raw materials required for viral replication, which indirectly suppresses viral proliferation. In vitro studies have demonstrated that pralatrexate effectively inhibits SARS-CoV-2 replication at extremely low concentrations in Vero and human lung epithelial Calu-3 cells, with inhibitory effects superior to those of remdesivir [227,228,229]. Furthermore, mechanistic studies have revealed that pralatrexate may exert antiviral effects not only through antimetabolic actions but also via direct inhibition of viral targets such as the RdRp of SARS-CoV-2, exhibiting multi-target, broad-spectrum antiviral potential [229]. However, large-scale clinical trial data supporting its safety and efficacy in COVID-19 or other coronavirus infections are currently lacking, and its clinical translation is constrained by adverse effects such as myelosuppression [230]. Future research directions include dose optimization, combination therapy regimens, and exploratory applications in the veterinary field.

Metformin, as a classic biguanide antihyperglycemic agent, has demonstrated potential for drug repurposing in anti-coronavirus research in recent years. Its mechanisms of action primarily involve multi-pathway metabolic modulation: activation of the AMP-activated protein kinase (AMPK) pathway suppresses glycolysis and lipid synthesis, thereby restricting the supply of energy and nucleotides required for viral replication; concurrently, it inhibits NF-κB-mediated inflammatory responses to attenuate the cytokine storm while enhancing antiviral immunity [231,232]. In vitro experiments demonstrated that metformin inhibited SARS-CoV-2 in Calu-3 and Caco-2 cell lines with half-maximal inhibitory concentrations (IC_50_) of approximately 0.4 mM and 1.4 mM, respectively, reducing infectious titers by up to 99% without significant cytotoxicity [233]. Metformin also exhibits promising therapeutic potential in animal coronavirus infection models. Research has demonstrated that infection with porcine enteric *Alphacoronavirus* (PEAV) relies on host lipid metabolism to promote viral replication, whereas metformin can reduce viral replication through AMPK-mediated suppression of lipid droplet formation [234]. Collectively, metformin possesses the dual functions of metabolic reprogramming and immune regulation, and investigations into its application in COVID-19 and animal coronavirus infections provide a solid foundation for drug repurposing.

Rapamycin is a canonical mTORC1 inhibitor clinically applied for allograft rejection prophylaxis and the treatment of autoimmune diseases. Its anti-coronavirus mechanisms are multifaceted and complicated. On one hand, mTORC1 suppression blocks virus-dependent protein biosynthesis, reduces proinflammatory cytokines, including IL-6 and IL-2, to alleviate cytokine storm, and facilitates autophagy-mediated clearance of virions [235,236]. On the other hand, a 2022 study demonstrated that rapamycin and everolimus trigger lysosomal degradation of IFITM2/3 via TFEB nuclear translocation, thereby facilitating SARS-CoV-2 cellular entry and implying a potential proviral effect rather than antiviral activity [237]. In vitro investigations have shown that pre-infection pharmacological inhibition of the mTOR pathway achieves approximately 60% suppression against MERS-CoV infection [238]. Preclinical and animal model studies have validated that rapamycin mitigates COVID-19-associated inflammatory storm and organ injury via immunomodulation and restrained IL-6 secretion [235,239]. Clinically, rapamycin and its rapalog derivatives augment COVID-19 vaccine-elicited memory T-cell responses, holding promise for improving vaccine efficacy in immunocompromised populations [240]. In preclinical assays targeting animal coronaviruses, rapamycin-induced autophagy restricts the infectivity of PEDV in porcine intestinal epithelial cells [241]. Collectively, the clinical antiviral use of rapamycin requires careful risk–benefit evaluation to balance its immunomodulatory advantages against the inherent risk of facilitating viral cellular entry.

## 4. Core Challenges and Future Research Directions for Cross-Species Drug Repurposing

### 4.1. Core Challenges in Cross-Species Drug Repurposing

Cross-species drug repurposing serves as a cost-effective and rapid-response strategy for both public health prevention of human coronavirus infections and disease control in livestock and poultry. However, this approach still faces two core challenges. 

Species-specific pharmacokinetic (PK) differences exert substantial influence on drug development and cross-species drug repurposing. Drug-metabolizing enzymes (such as the cytochrome P450 family) exhibit significant interspecies differences in expression levels and enzymatic activity, which directly alter the absorption, distribution, metabolism, and excretion (ADME) processes of drugs, ultimately resulting in marked differences in half-life, clearance, and pharmacological efficacy across species [242,243,244,245]. Nirmatrelvir is primarily metabolized by the CYP3A4 enzyme system. Interspecies differences in enzymatic activity give rise to marked divergence in quantitative pharmacokinetic profiles. The oral bioavailability of nirmatrelvir in cynomolgus monkeys is merely 8.5% with a half-life of 0.8 h, whereas its human half-life can reach ~6 h when co-administered with ritonavir. Consequently, at equivalent doses, systemic exposure in monkeys is substantially lower than in humans, necessitating higher doses or repeated administration to achieve efficacious unbound concentrations in preclinical studies [246]. Consequently, PK data obtained from animal experiments possess inherent limitations when extrapolated to humans, particularly for drugs undergoing highly species-specific metabolism, and vice versa. Beyond PK disparities, species-specific variations in viral targets also constrain the efficacy of cross-species therapeutic application. Although core functional targets of coronaviruses exhibit a degree of evolutionary conservation, key functional sites within target proteins (such as drug-binding pockets and enzymatic active centers) in viruses derived from different host species may still harbor amino acid mutations [247]. As exemplified by the veterinary clinical agent GC376, this compound exhibits potent inhibitory activity against the 3CLpro of FIPV, with a Ki of 2.1 nM. In contrast, its Ki against wild-type SARS-CoV-2 3CLpro rises to 40 nM, representing an approximately 20-fold reduction in binding affinity and a marked attenuation of cross-species antiviral efficacy [248]. Such variations can alter the binding conformation or affinity between drugs and their targets, thereby causing agents effective in one species to exhibit significantly reduced inhibitory activity against coronaviruses in another species.

Cross-species drug repurposing also confronts dual obstacles arising from regulatory system barriers and the high costs of clinical trials. At the regulatory level, the human drug and veterinary drug evaluation systems of major global regulatory agencies remain independent, and no dedicated approval pathway has yet been established specifically for cross-species drug repurposing [249]. Furthermore, veterinary drugs must strictly adhere to maximum residue limit (MRL) standards based on scientific risk assessment; however, certain repurposed human antiviral drugs (such as ribavirin) possess potent teratogenicity and genotoxicity, and their metabolic residues in animals are difficult to reconcile with MRL criteria, directly restricting the translational application of such agents to veterinary medicine. For instance, ribavirin inhibits PEDV in vitro but is prohibited in food-producing animals in China due to residue hazards and teratogenicity. Likewise, clinical data for GC376—a standard therapy for feline infectious peritonitis—cannot be directly extrapolated to human coronavirus indications, necessitating de novo non-human primate challenge studies and human clinical trials. At the clinical trial level, cross-species research faces practical, operational and ethical constraints. On one hand, per-animal trial costs for large livestock are high, and substantial inter-individual variability makes it difficult to conduct large-sample randomized controlled trials (RCTs) analogous to those performed in humans; on the other hand, the dual requirements of human subject protection and animal ethics preclude the design of controlled studies simultaneously encompassing both human and animal subjects, thereby rendering direct validation of drug efficacy in blocking cross-species coronavirus transmission impossible. Consequently, researchers must rely on indirect inferences derived from in vitro cellular experiments or interspecies in vivo studies, which diminishes the reliability of conclusions.

In summary, interspecies pharmacokinetic variations and low conservation of viral targets represent the core scientific obstacles limiting the cross-species application of repurposed anti-coronavirus drugs. In addition, separated human and veterinary drug regulatory systems, together with the high costs of cross-species trials, further hinder clinical translation. Together, these factors form the critical bottleneck constraining the transition of such repurposed agents from basic research to multispecies clinical application.

### 4.2. Future Research Directions and Clinical Translation Strategies for Cross-Species Drug Repurposing

To address core bottlenecks such as species differences and target variations, and to accelerate the cross-species repositioning of anti-coronavirus agents, this review proposes a systematic strategy encompassing three dimensions: technology empowerment, model innovation, and systemic synergy.

First, in the dimension of technology empowerment, we aim to construct an AI-empowered intelligent cross-species drug screening system. The core of this system lies in integrating multidimensional “coronavirus–drug–host” data and enhancing efficiency through two major innovations: (i) developing customized molecular docking algorithms tailored to species-specific target variations, thereby substantially improving the accuracy of virtual screening; and (ii) establishing deep learning–based pharmacokinetic/pharmacodynamic (PK/PD) prediction models that precisely forecast key PK parameters in critical species—including humans, swine, felines, and bovines—by dissecting interspecies differences in metabolic enzyme activity. For example, marked interspecies divergence exists among CYP3A isoforms: human drug metabolism is predominantly mediated by CYP3A4, whereas porcine liver relies on CYP3A29 and related isoforms (e.g., CYP3A22, CYP3A46). Nirmatrelvir undergoes hepatic metabolism via CYP3A enzymes, so extrapolating livestock dosing purely from human metabolic data introduces substantial discrepancies. By contrast, an integrated AI-PBPK platform can aggregate multi-species metabolic datasets to precisely predict the in vivo pharmacokinetic profiles of nirmatrelvir in livestock, addressing the limitation that human pharmacological parameters cannot be directly applied to veterinary dosage regimen design [250].

Second, in the dimension of model breakthroughs, to address the bottleneck of strong species specificity and low predictive efficiency for clinical translation inherent in traditional infection models, we aim to develop a new generation of cross-species-compatible infection evaluation models. Currently, non-animal methods represented by organ-on-a-chip and human cell–based models are receiving increasingly explicit policy support; for instance, the United States is actively promoting their substitution for animal experiments through legislation such as the FDA Modernization Act 2.0, while China has incorporated these technologies into the key technological scope of digital and intelligent pharmaceutical development under its Implementation Plan for Digital and Intelligent Transformation of the Pharmaceutical Industry. Guided by this policy orientation, this review focuses on two directions: (i) developing organoid-based cross-species high-throughput screening platforms, utilizing pluripotent stem cells from susceptible species (including humans, swine, and felines) to construct respiratory and intestinal organoids, thereby establishing a parallel human-derived and animal-derived infection assessment system capable of simultaneously evaluating antiviral activity, cross-species metabolic characteristics, and potential toxicity; and (ii) developing intelligent humanized animal models supporting multispecies evaluation, with the goal of assessing synergistic therapeutic efficacy within a single living system, thereby transcending species limitations and providing a basis for precision dosing regimen design and molecular mechanism elucidation. Chau et al. established human ACE2 transgenic pigs susceptible to SARS-CoV-2 that develop authentic COVID-19-like disease with fever, respiratory distress, and lung immunopathology, offering superior physiological fidelity over conventional rodent platforms [251]. Scaling such platforms toward multispecies immune reconstitution would enable parallel cross-species therapeutic evaluation, representing the next frontier in precision veterinary medicine.

Finally, in the dimension of systemic synergy, we propose the construction of a human–veterinary dual-track collaborative drug research and development system to accelerate translational outcomes. The core of this system is to promote the establishment of a cross-species drug repurposing consortium jointly participated in by pharmaceutical enterprises, leading research institutions, and drug regulatory agencies. The alliance’s missions concentrate on two priorities: (i) achieving deep resource sharing by systematically integrating approved human and veterinary drug libraries, harmonizing relevant regulatory barriers, and constructing physical and virtual screening libraries dedicated to cross-species coronavirus drug repurposing; and (ii) establishing unified data platforms by jointly constructing and maintaining a globally shared coronavirus drug repurposing database, formulating standardized data specifications, and aggregating and updating comprehensive experimental data for drugs across different species in real time. This collaborative system maximally avoids redundant research and development, enabling drug molecules whose safety has been validated in any single species to be rapidly redirected toward clinical application in another species, thereby establishing an efficient integrated laboratory-to-farm-to-clinic translational pathway.

**Figure 4:**
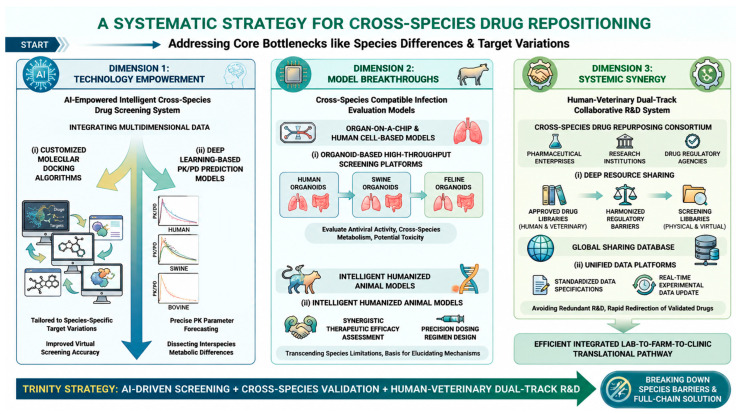
A systematic strategy for cross-species drug repositioning. The trinity framework addresses core bottlenecks of species differences and target variations through three dimensions: (i) technology empowerment: an AI-driven cross-species drug screening system with customized molecular docking and deep learning-based pharmacokinetic/pharmacodynamic (PK/PD) prediction; (ii) model breakthroughs: cross-species-compatible infection evaluation models, including organoid-based screening platforms (human, swine, feline) and intelligent humanized animal models; and (iii) systemic synergy: a human–veterinary dual-track R&D system via cross-species consortiums, shared drug libraries, and unified global data platforms.

In summary, this study proposes a trinity strategy integrating “AI-driven intelligent screening, cross-species model validation, and human–veterinary dual-track R&D”. This multi-technology and multi-stakeholder framework systematically breaks species-specific barriers and provides a full-chain solution for efficient cross-species repurposing of anti-coronavirus drugs (Figure 4). This integrated system is expected to substantially enhance drug development and translational efficiency, furnish critical technological support for the rapid response to emerging zoonotic viral outbreaks, and carry significant strategic importance for safeguarding public health security and promoting the healthy development of the livestock industry.

## 5. Conclusions

**Figure 5:**
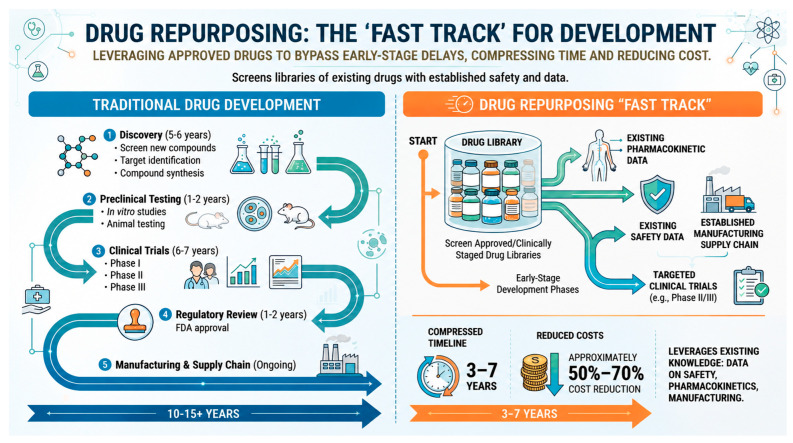
Drug repurposing as a fast-track strategy for antiviral development. Drug repurposing acts as a fast-track strategy, leveraging existing drug safety and manufacturing data to compress the development timeline from 10 to 15+ years to 3–7 years, while reducing costs by 50–70%.

Emerging and re-emerging viral diseases underscore the persistent threat posed by viral cross-species transmission, and the prevention and control of viral infections necessitate an integrated broad-spectrum strategic framework incorporating vaccines, antibodies, direct-acting antivirals, and host-directed interventions. Within this framework, drug repurposing—leveraging its advantages of rapid response, controllable costs, and reusable safety datasets—has emerged as one of the most clinically translatable platform technologies for responding to sudden infectious disease outbreaks (Figure 5).

Using coronaviruses as a paradigm, this review systematically elucidates the broad-spectrum mechanisms and cross-species translational pathways of drug repurposing (Supplementary Table S1). At the virus-targeting level, conserved enzymes including 3CLpro, RdRp, and PLpro provide a molecular basis for nucleoside analogues and protease inhibitors to achieve pan-variant and cross-genera antiviral coverage. At the host-targeting level, entry-dependency factors such as ACE2 and TMPRSS2, alongside evolutionarily conserved metabolic pathways including glycolysis and pyrimidine synthesis across mammals, endow receptor antagonists, protease inhibitors, and metabolic modulators with the potential for human and veterinary shared application. Of particular importance, host-directed strategies fundamentally circumvent the issue of drug resistance by establishing a “host defense line” that is insensitive to viral mutations.

Nevertheless, cross-species translation continues to face three major challenges: species-specific pharmacokinetic disparities, inter-genera variations in viral targets, and the fragmentation between human and veterinary drug regulatory systems. Future breakthroughs must be pursued along three dimensions: technological empowerment through AI-driven cross-species drug prediction and parallel organoid screening; model innovation through intelligent interspecies-compatible infection models; and systemic synergy through human–veterinary dual-track regulatory consortia and globally shared databases. These initiatives will not only accelerate the clinical translation of anti-coronavirus therapeutics but also, more importantly, pre-position a rapidly deployable broad-spectrum antiviral platform for responding to future emerging viral outbreaks.

## Data Availability

No new data were created or analyzed in this study. Data sharing is not applicable to this article.

## Supplementary Materials

The following supporting information can be downloaded at: https://www.mdpi.com/article/10.3390/v18070804/s1, Table S1: Summary of Repurposed Drugs with Broad-Spectrum Anti-Coronavirus Effects: Molecular Mechanisms and Translational Constraints.
