# Supplementary material for: Drug Repurposing as a Broad-Spectrum Strategy Against Coronaviruses: Frontiers in Mechanisms and Clinical Translation
Source: Viruses. 2026 Jul 21;18(7):804. doi: 10.3390/v18070804 (PMC13431539; doi:10.3390/v18070804)
Supplement: Supplementary file 1 [file viruses-18-00804-s001.zip › viruses-4409770-supplementary.pdf]

Table S1. Summary of Repurposed Drugs with Broad-Spectrum Anti-Coronavirus Effects: Molecular Mechanisms and Translational Constraints

Drug Name	Basic Information	Core Anti-coronavirus Mechanisms	Safety and Limitations
Ursodeoxycholic acid (UDCA)	An endogenous bile acid indicated for cholestatic liver diseases and gallstone dissolution.	It inhibits the FXR signaling pathway to downregulate ACE2 expression[58].	Its prophylactic effect requires further clinical validation; its mechanism is well-defined, but inconsistent findings exist among distinct studies.
Lactoferrin (LF)	A natural iron-binding glycoprotein; oral and intranasal administration is under investigation for prophylaxis and adjunctive therapy.	LF binds to HSPGs to block the initial attachment between viral S-protein and host cells[26]; it directly interacts with the RBD of S-protein to hinder S-protein–ACE2 binding, and suppresses the catalytic activity of RdRp[63, 64].	It has a favorable safety profile; large-scale clinical trials are required to confirm its efficacy and applicable scenarios.
Statins	Conventional lipid-lowering agents extensively studied for drug repurposing against coronavirus.	It inhibits HMG-CoA reductase to reduce membrane cholesterol and disrupt lipid rafts; it downregulates CD147 and modulates autophagic flux[68].	Multiple pharmacological mechanisms with prominent clinical relevance; their efficacy against animal coronaviruses awaits experimental verification.
Methyl-β-cyclodextrin (MβCD)	A promising repurposing candidate and commonly used pharmaceutical excipient.	MβCD depletes membrane cholesterol and disrupts lipid rafts to block virus-receptor interaction[75]; additionally, it can serve as a pharmaceutical carrier to improve the therapeutic efficacy of combined agents.	High biosafety plus inherent advantages as a delivery carrier; large-scale human clinical trials are needed to verify in vivo efficacy and safety.

Chloroquine (CQ)/Hydroxychloroquine (HCQ)	Approved for malaria and autoimmune disorders and previously evaluated for COVID-19 treatment.	CQ/HCQ elevate endosomal and Golgi pH, blocking viral entry and glycosylation, and inhibiting S protein–ganglioside interaction[78, 80].	Effective in vivo concentrations can hardly be achieved without exceeding safe doses, accompanied by obvious cardiotoxicity.
Enoxaparin	A low-molecular-weight heparin clinically used for anticoagulation during COVID-19 treatment.	Enoxaparin binds to the RBD of SARS-CoV-2 S protein and ACE2, blocking their interaction and viral entry.[87]; it potentiates the inhibitory effect of α1-antitrypsin against TMPRSS2[88].	Antiviral evidence is mainly obtained from in vitro mechanistic research.
Camostat mesylate	Approved in Japan in 2001 for pancreatitis; an oral serine protease inhibitor.	It inhibits TMPRSS2 to block S-protein activation and virus-host membrane fusion via stable binding to active-site residues of TMPRSS2[91].	In vitro and animal tests suggest broad-spectrum antiviral potential; clinical efficacy remains unconfirmed and further coronavirus animal experiments are necessary.
Amantadine	A canonical ion channel blocker approved for influenza prophylaxis/treatment and Parkinson’s disease.	It downregulates cathepsin L to hamper S-protein cleavage, inhibits the ion channel activity of E protein and ORF10, and potentially disrupts virus-receptor binding[99, 100].	Multiple-target antiviral activity in vitro but limited in vivo potency; lacking animal and clinical data for translational assessment.

E-64d	A cysteine protease inhibitor.	It inhibits cathepsin L to block viral endosomal escape and suppresses 3CLpro-mediated polyprotein cleavage[103-105].	Research is restricted to molecular, cellular and preclinical animal studies; no available human clinical trial data.
Nafamostat mesylate	A marketed serine protease inhibitor applied for anticoagulation during hemopurification.	Inhibit the host cell surface protease TMPRSS2, thereby blocking spike protein-mediated membrane fusion and cellular entry of coronaviruses[107-109].	Favorable potency in in vitro, animal and preliminary clinical research; definitive clinical benefits remain unclear pending high-quality trials.
Remdesivir	Nucleoside prodrug authorized by the FDA for COVID-19 in 2020.	Metabolized into GS-443902, which incorporates into viral RNA and triggers delayed chain termination[113].	Controversial clinical efficacy; inefficient intracellular transformation into active GS-441524.
Molnupiravir	Oral nucleoside prodrug approved for COVID-19 in 2021.	Converted into NHC-TP to induce widespread viral mutagenesis and lethal error catastrophe[115].	Clinically available in humans but insufficiently validated for animal administration.
Azvudine	Synthetic nucleoside analog originally developed against HIV.	Its active metabolite FNC-TP inserts into viral RNA to interrupt genome synthesis, with dual anti-HIV and anti-coronavirus activity[130].	Current data are derived mostly from small-cohort trials; large-scale clinical validation is required.
Suramin	Centurial clinical drug initially approved for African trypanosomiasis and onchocerciasis.	Inhibits the RdRp, S protein and N protein of coronavirus[133-135].	Clear in vitro mechanisms but insufficient in vivo pharmacodynamic and safety data; limited research across human and animal coronaviruses.

GC373/GC376	Originally developed targeting feline coronavirus 3CLpro; GC376 is clinically available for feline infectious peritonitis.	Covalently binds to the catalytic Cys145 of 3CLpro and blocks polyprotein proteolysis[141, 143].	Unavailable for human clinical application; solid preclinical animal foundation requires further translational expansion.
Baicalein	Core bioactive constituent of Shuanghuanglian oral liquid; non-covalent non-peptidic 3CLpro inhibitor.	Inserts between catalytic His41 and Cys145 of 3CLpro to hinder substrate access to the active pocket[147].	A promising lead compound waiting for pharmaceutical development.
Boceprevir	Approved antiviral agent against hepatitis C virus.	Occupies the catalytic pocket of 3CLpro to inhibit polyprotein maturation via protease cleavage blockade[142].	Emerging drug resistance restricts clinical translation requiring deeper investigation.
Chlorogenic acid	Bioactive component from honeysuckle; approved as animal feed additive in 2019.	It may suppress 3CLpro activity, interrupt S-protein–ACE2 binding and modulate innate immune signaling cascades to exert anti-coronavirus activity[152, 153].	Research confined to in vitro and animal models; additional preclinical verification is needed.
Carmofur	Antineoplastic agent with novel derivatives developed targeting viral 3CLpro.	Covalently binds to catalytic Cys145 of 3CLpro via carbonyl group, with aliphatic tail occupying hydrophobic S2 subsite to block polyprotein cleavage[156].	Suboptimal inhibitory efficiency in cellular models with reduced activity under reductive conditions; no animal or human clinical data.
Disulfiram	Classic alcohol-aversion drug under repurposing research.	Covalently modifies key cysteine residues of 3CLpro to block polyprotein cleavage[160].	Supporting evidence is limited to in vitro experiments and retrospective clinical analyses.

Ebselen	Organoselenium compound previously evaluated as antioxidant and cytoprotective agent.	Inhibits 3CLpro and PLpro, acts as zinc ejector against viral zinc-finger replication domains and synergizes with remdesivir[161, 163, 164].	Validated only via in vitro enzymology, cellular and structural biological studies.
Nitazoxanide	Anti-parasitic drug approved in 2002 with broad-spectrum antiviral property.	It may function by boosting innate immunity and disrupting viral polypeptide processing[166].	Satisfactory safety profile; human and animal applications remain in exploratory phase.
Niclosamide	Clinically available antihelminthic agent.	It is speculated to block viral entry, internalization and RNA synthesis via suppressing endosomal acidification[172].	Low oral bioavailability necessitating optimized drug delivery formulations.
Tocilizumab	Humanized anti-IL-6R monoclonal antibody approved for rheumatoid arthritis.	Blocks IL-6-receptor interaction to suppress inflammatory cascades and alleviate cytokine storm[181].	Heterogeneous clinical efficacy; lacking direct in vivo evidence from animal experiments.
Anakinra	Recombinant human IL-1 receptor antagonist for autoinflammatory disorders.	Neutralizes IL-1α/IL-1β and inhibits NLRP3 inflammasome-triggered inflammatory responses[179].	Confirmed clinical efficacy in humans; broad-spectrum immunomodulatory potential in animals remains unstudied.
Fluvoxamine	SSRI-type antidepressant for obsessive-compulsive disorder and depression.	Activates σ-1 receptor to mitigate endoplasmic reticulum stress and inflammation, alongside suppression of platelet aggregation and viral endocytosis[191, 193].	Well-tolerated in humans; no supporting preclinical animal data.

Baricitinib	Oral JAK1/JAK2 inhibitor approved for rheumatoid arthritis.	Suppresses JAK-STAT cascade to relieve cytokine storm and inhibits AAK1/GAK to hinder viral endocytic uptake[193, 201].	Potential delayed viral clearance and elevated secondary infection risk; personalized immune status assessment is required.
Dexamethasone	Classic glucocorticoid with potent anti-inflammatory and immunoregulatory effects.	Restrains excessive immune overactivation and cytokine storm to reduce ARDS and pulmonary injury via regulating neutrophil subsets[206, 207].	Increased secondary infection risk; rigorous indication screening and risk-benefit evaluation are mandatory.
Cetaben	Classic ACAT (SOAT) inhibitor originally developed as a cholesterol-lowering drug.	inhibits ACAT to disrupt cellular cholesterol homeostasis and lipid raft formation[212, 215].	antiviral efficacy is merely confirmed via in vitro assays, no valid in vivo animal or human clinical data available for coronavirus therapy.
Leflunomide	Isoxazole immunomodulator and anti-rheumatic agent for routine clinical use.	Inhibits DHODH to terminate de novo pyrimidine synthesis and blocks JAK/STAT/NF-κB pathways to lower pro-inflammatory cytokines[222, 223].	Dual metabolic-inhibitory and immunomodulatory activity with promising cross-species potential yet limited efficacy for severe cases.
Pralatrexate	FDA-approved dihydrofolate reductase inhibitor.	Interrupts viral nucleotide precursor supply via restraining folate metabolism and potentially inhibits RdRp directly[228, 229].	Myelosuppression limits clinical translation; further dose adjustment and combination therapy exploration are needed.
Metformin	First-line biguanide anti-diabetic drug widely prescribed clinically.	Activates AMPK to suppress glycolysis/lipogenesis, inhibits NF-κB-mediated inflammation and enhances host antiviral immunity[231].	Therapeutic outcomes vary by dosage, administration timing and patient population with favorable cross-species development prospect.

Rapamycin	Classic mTOR inhibitor used for post-transplant rejection prophylaxis and autoimmune diseases.	Blocks mTOR pathway to suppress viral protein biogenesis, modulates immunity against cytokine storm and promotes autophagy-mediated viral clearance[235, 236].	Dual antiviral and immunoregulatory benefits without large-scale prospective clinical proof-of-concept data.
